# Left ventricle remodelling by double-patch sandwich technique

**DOI:** 10.1186/1749-8090-2-10

**Published:** 2007-01-31

**Authors:** Ernesto Tappainer, Vinicio Fiorani, Nicola Pederzolli, Jacopo Manfredi, Andrea Nocchi, Mario Zogno

**Affiliations:** 1 Cardiac Surgery Unit, "Carlo Poma" Hospital, Viale Albertoni 1, 46100 Mantua, Italy

## Abstract

**Background:**

The sandwich double-patch technique was adopted as an alternative method for reconstruction of the left ventricle after excision of postinfarction dysfunctional myocardium to solve technical problems due to the thick edges of the ventricular wall.

**Methods:**

Over a 5-year period, 12 of 21 patients with postinfarction antero-apical left ventricular aneurysm had thick wall edges after wall excision. It was due to akinetic muscular thick tissue in 6 cases, while in the other 6 with classic fibrous aneurysm, thick edges remained after the cut of the border zone. The ventricular opening was sandwiched between two patches and this is a technique which is currently used for the treatment of the interventricular septum rupture. In our patients the patches are much smaller than the removed aneurysm and they were sutured simply by a single row of single stitches. However, in contrast to interventricular septum rupture where the patches loosen the tension of the tissues, in our patients the patches pull strongly and restrain the walls by fastening their edges and supporting tight stitches. In this way they could narrow the cavity and close the ventricle.

**Results:**

The resected area varied from 5 × 4 to 8 × 8 cm. Excision was extended into the interventricular septum in 5 patients, thus opening the right ventricle. CABG was performed on all patients but two. Left ventricular volumes and the ejection fraction changed significantly: end-systolic volume 93.5 ± 12.4 to 57.8 ± 8.9 ml, p < 0.001; end-diastolic volume 157.2 ± 16.7 to 115.3 ± 14.9 ml, p < 0.001; ejection fraction 40.3 ± 4.2 to 49.5 ± 5.7%, p < 0.001. All patients did well. One patient suffered from bleeding, which was not from the wall suture, and another had a left arm paresis. The post-operative hospital stay was 5 to 30 days with a mean 10.5 ± 7.5 days/patient. At follow-up, 9 to 60 months mean 34, all patients were symptom-free. NYHA class 2.5 ± 0.8 changed to 1.2 ± 0.4, p < 0.001.

**Conclusion:**

The double-patch sandwich technique (bi-patch closure) offers some advantages and does not result in increased morbidity and mortality. In the case of excising a left ventricular aneurysm, this technique in no way requires eversion of the edges, felt strips, buttressed and multiple sutures, all of which are needed for longitudinal linear closure. Moreover, it does not require purse string sutures, endocardial scar remnant to secure the patch or folding the excluded non-functional tissue, all of which are needed for endoventricular patch repair.

## Background

Cooley linear closure [1] and Dor endoventricular patch plasty [2] are commonly used techniques of reconstruction after post-infarction left ventricular aneurysm (LVA) resection. However, in cases of thick and soft edges of the ventricular wall, we adopted an alternative sandwich double-patch technique for reconstruction and LV closure. This occurred when the resection fell on the thick border zone of a classic fibrous diskinetic aneurysm, or during excision of thick muscular akinetic aneurysms. Medical therapy such as reperfusion of the infarct-related artery and angiotensin converting enzyme (ACE) inhibitor therapy has changed the evolution of the infarct area to scarred LVA [3]. This approach has increased the occurrence of partially damaged muscle, which appears as a thick dysfunctional area around a thin focal scar. Buckberg proposed that the definition of aneurysm should include this non-functional myocardium, which has become a large component of LVA and has to be resected [3]. In these patients, resection leaves thick soft wall edges which cannot be easily everted to perform the classic Cooley longitudinal buttressed suture, but at the same time the endocardium is too weak to allow a firm suture of the single patch to perform the Dor exclusion of the aneurysm.

In recent years, along with the classic techniques, we used the sandwich double-patch method of LV reconstruction in 12 patients who showed thick wall edges of the ventricle. Results were good for early and long term.

## Methods

### Patients

The double patch technique was chosen for the first time as a consequence of a mistake: an excessive excision of the lateral wall during a fibrous LVA resection. The excellent result led us to perform this technique along with traditional procedures. A comparative study between techniques is beyond the aim of this paper. Written informed consent for aneurysm resection was obtained from all patients with dyskinetic LVA, while patients with akinetic muscular LVA were informed about the possibility of the resection, which was carried out only if a central thin scar confirmed the transmurality of the infarction. Recently the Ethics Committee of our Hospital required us to inform patients about this technique of reconstruction.

The sandwich double-patch technique was used on 12 patients with antero-apical myocardial infarction due to left anterior descending (LAD) artery closure (Table 1) between December 2000 and December 2005. In the same period 9 patients had 5 Cooley and 4 Dor procedures of LV reconstruction. In the study group there were 5 male and 7 female patients aged 57 to 76 years (67 ± 5.8 mean ± SD). Patients had single vessel coronary disease in 3 cases. Myocardial infarction (MI) occurred 8 days to 16 years (mean 40.5 ± 68.7 months) before the operation. 6 patients showed akinetic LVA aspect at cardiac imaging while the other 6 had classic dyskinetic aneurysm. 3 patients had a silent MI. None had angioplasty. 2 patients only received thrombolysis and one of them had successful reperfusion with patent LAD and TIMI grade 3 flow. Nevertheless, this patient developed a muscular akinetic LVA. 1 patient was on cardiogenic shock due to a very early antero-apical enlargement and impending cardiac rupture. None had significant mitral insufficiency. All but one were on ACE-inhibitor therapy. 1 patient was on chronic dialytic treatment. Myocardial viability was assessed by Echo in all patients, 3 of them had a SPECT-TC99 analysis for confirmation. LV thrombosis was suspected in 5 patients (Table 1). Body surface area, preoperative ejection fraction (EF), end-systolic volume and end-diastolic volume (EDV) are listed and compared with postoperative data in the third table.

**Table 1:** Preoperative characteristics

Pt	Gender	Age	Symptoms	NY HA class	Vessel disease	Infarct-surgery delay	Thrombolysis	TIMI grade LAD	ACE inhib therapy	LV thrombus suspected	Viability test
1	F	76	A+D+Ar	2	2	6 mo	No	1	Yes	Yes	Echo
2	F	57	A	2	3	2 yr+2 mo	Yes	0	Yes	No	Spect-tc
3	M	61	None	-	3	1 mo	Yes	3	Yes	No	Echo
4	M	65	D, Rf	3	2	7 yr+5 mo	No	1	No	Yes	Echo
5	F	68	D	3	3	Silent	No	0	Yes	No	Echo
6	F	71	A+D	2	2	3 yr	No	0	Yes	No	Spect-tc
7	F	61	Shock	4	1	8 days	No	0	Yes	No	Echo
8	M	75	D	3	3	3 mo	No	0	Yes	Yes	Spect-tc
9	M	67	A+E	3	2	Silent	No	0	Yes	Yes	Echo
10	F	69	D	3	1	10 yr	No	1	Yes	No	Echo
11	M	63	A+E	2	3	16 yr	Yes	1	Yes	Yes	Echo
12	F	71	D	2	1	Silent	No	1	Yes	No	Echo
mean ± sd		67 ± 5.8		2.5 ± 0.8		40.5 mo ± 68.7					

A: angina; D: dyspnea; Ar: arrhytmias; Rf: renal failure; E: embolism.

### Technique

Resection of LVA was performed on normothermic CPB (Oxygenator Avant D903 Sorin Group, Mirandola, Italy) and a beating heart to guide excision by digital palpation. CABG was also performed on a beating heart whenever possible. LAD artery was treated if usable and not involved in the resection. Mammary artery graft was used in younger patients and on most developed diseased branches but not on the LAD artery.

**Figure 1:**
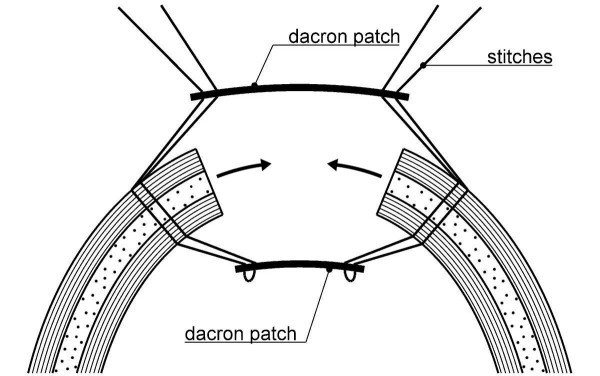
**Drawing of the two Dacron patches**. During the closure, for each stitch the external patch acts as a pulley to attract the walls towards the centre of the resected area

Two types of patient were treated, all with thick wall edges after the resection. These were patients with akinetic muscular aneurysm and patients with classic dyskinetic aneurysm when the cut was carried on the thick border zone. A patch was applied to both the internal and external surfaces of the ventricular wall in order to restrain the ventricular opening, to reduce the cavity and to close the residual opening (Figure 1). In cases of irregular shape the patches can imitate the resected area and reduce it in size without distorting the geometry of the chamber.

**Figure 2:**
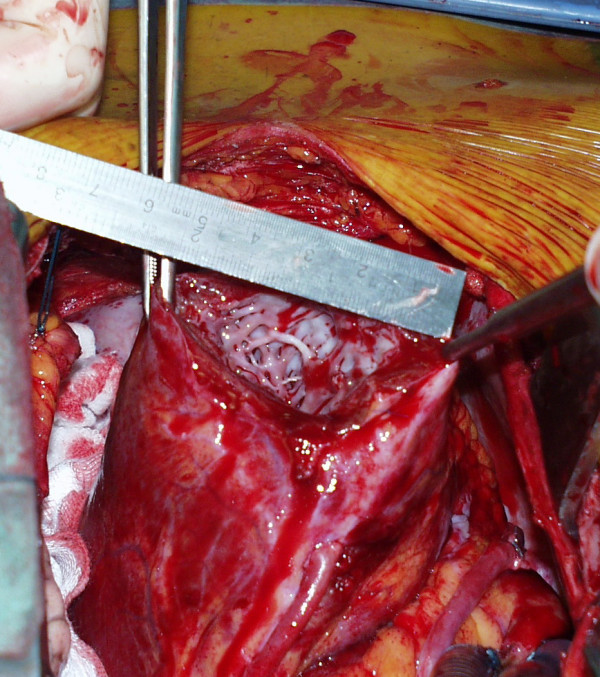
**Patient n°3: antero-apical excision for akinesia without septal involvement**. The thick cut surface is somewhat bleeding because the heart was beating

**Figure 3:**
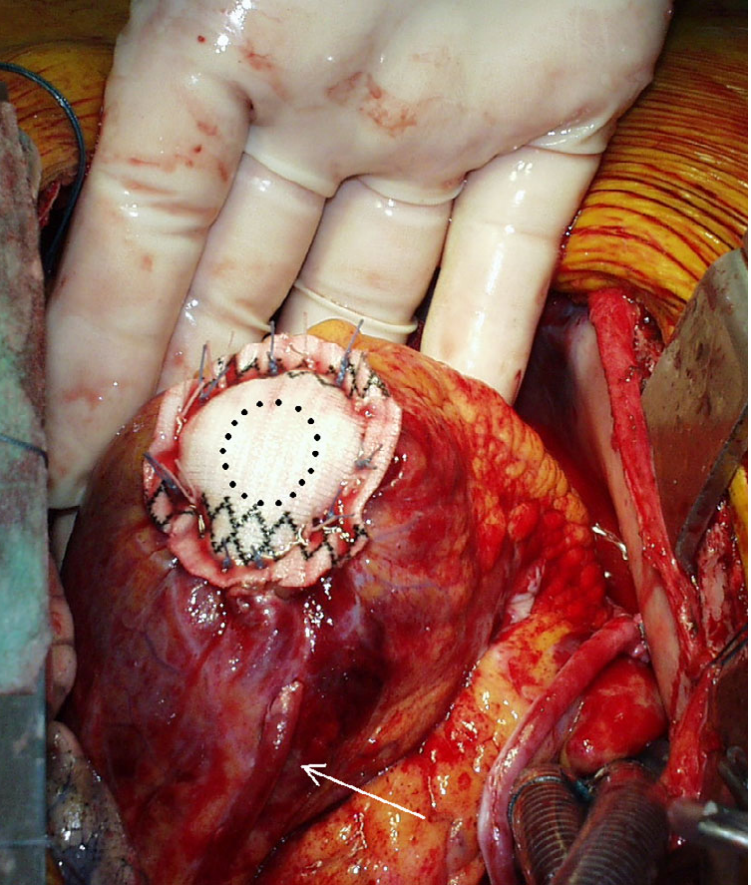
**Patient n°3: final aspect**. Dotted circle: size of the remaining hole. Arrow: vein graft on the LAD artery (IMA was grafted on a large obtuse marginal branch).

The patches were tailored from a Dacron aortic vascular prosthesis (Gelseal Triaxial, Sulzer Vascutek Ltd, Glasgow, Scotland) which was opened longitudinally. An additional heterologous pericardial patch (Peripatch, Sorin Biomedica, Saluggia, Italy) was used in 3 patients to cover the inner surface of the internal Dacron patch and to reduce the risk of thromboembolism. The suture was a single row of single, double-armed, woven non-resorbable stitches passing through the myocardium about 1 cm from the edge, from the inner to the outer patch. In cases of greater involvement of the interventricular septum, a reduction treatment of the septum was carried out before inserting the patches. Purse string sutures, felt strips, oversewing sutures, eversion of the wall edges and folding tissue were carefully avoided (Figure 2, 3).

### Statistical analysis

Data were expressed as mean ± SD. Paired data were compared by two-tailed t-Student test.

## Results

The resected area ranged from 5 × 4 to 8 × 8 cm (mean 35.5 ± 12.1 cm^2^) in width. 6 patients had classic fibrous LVA. Another group of 6 patients showed a large akinetic area of near normal muscular tissue surrounding a small thin scar. The thin scar confirmed the transmurality of infarction in all patients with the exception of the 8 day infarction which showed an impending rupture. In 5 cases resection was extended to the interventricular septum so that the right ventricle was opened and its wall involved in the reconstruction.

**Figure 4:**
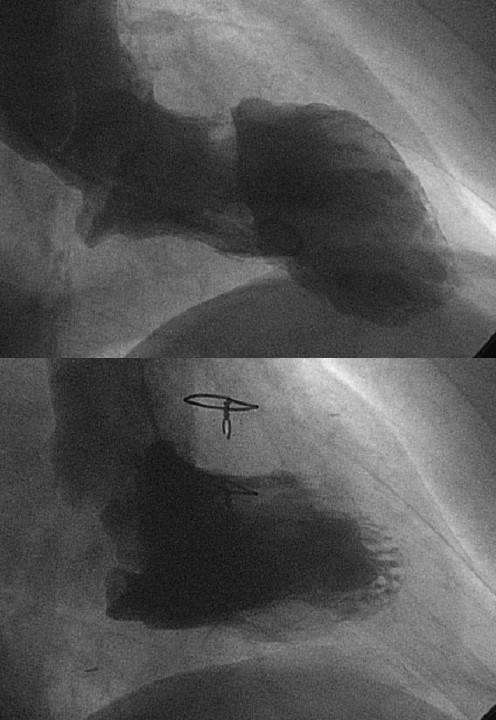
**Patient n°11: systolic shape at pre-post-operative angiography**. A satisfactory conical shape was achieved after antero-septal excision and reconstruction by double-patch technique.

CABG was performed in all but two patients. Five patients had 2 grafts, three patients had 3 grafts, one had single graft and one 5 grafts (mean 2.5 ± 1.0 grafts/patient). LAD artery was grafted in 4 cases only, because of full length occlusion or involvement in the resection. The internal mammary artery was harvested in 3 patients: it was put on LAD artery in one of them, while in two others it was put on the obtuse marginal branch, which was the most developed of the arteries needing a graft. 4 patients needed brief periods of aortic clamping for distal graft anastomosis. CPB time ranged from 76 to 178 min (mean 132.5 ± 28.2 min). All patients did well (Table 2). Inotropic support (Dopamine) was needed for 1 patient only. 1 patient required repeated re-exploration for bleeding, but he did not bleed from the LV wall suture and developed temporary pulmonary and renal failure. On the first day, 10 patients were discharged from ICU. The patient with preoperative IABP required another day for weaning. On the fifth day after surgery a female patient showed left arm paresis; she was not on anticoagulation therapy for fear of bleeding from the LV wall sutures. After this fifth patient, we introduced the use of a third additional heterologous pericardial patch (Peripatch, Sorin Biomedica, Saluggia, Italy) to cover the inner surface of the internal Dacron patch in the hope of preventing thromboembolism. It was used in 3 patients in whom LV thrombosis was found. Moreover, we introduced a moderate anticoagulation in the following patients. Mean postoperative hospital stay was 10.5 ± 7.5 days/patient (Table 2). Patients showed improvement in the left ventricular function, a reduction of the cavity (Table 3) and an optimal LV reshaping (Figure 3, 4) in almost all of them. Follow-up, by direct examination or telephone interview, ranged from 9 to 60 months (mean 34 ± 18). All patients were symptom-free and felt better so that NYHA class improved substantially (Table 3). The patient with left arm paresis has partially recovered. As a consequence of the better cardiac output the patient who was on chronic dialytic treatment has improved renal function and the treatment was stopped.

**Table 2:** Intra and postoperative data

Pt N°	LVA type	Resected area (cm)	LV thrombosis	Septum treatment	N° Bypass	IMA graft on	Clamp time (min)	CPB time (min)	Ino tropes	Complications	ICU stay (days)	Hosp stay (days)
1	Dys	6 × 5	Yes	-	2	-	-	123	-	-	1	6
2	Aki	6 × 5	-	-	2	-	-	124	-	-	1	7
3	Aki	5 × 4	-	-	3	OM	17	110	-	-	1	7
4	Aki	6 × 6	-	Yes	3	-	-	160	-	preop Rf	1	18
5	Aki	6 × 6	-	Yes	2	-	34	171	-	L arm paresis	1	5
6	Aki	5 × 4	-	-	2	OM	34	110	-	-	1	6
7	Aki	6 × 5	Yes	-	-	-	-	135	-	preop shock	3	17
8	Dys	7 × 6	-	-	3	-	56	140	-	Bl, Pf, Rf	25	30
9	Dys	8 × 8	Yes	Yes	2	-	-	134	-	-	1	6
10	Dys	8 × 5	Yes	-	1	LAD	-	129	-	-	1	6
11	Dys	8 × 6	-	Yes	5	-	82	178	-	-	1	11
12	Dys	7 × 6	-	Yes	-	-	-	76	Dop	-	1	7
mean ± sd		35.5 cm^2 ^± 12.1			2.5 ± 1.0			132.5 ± 28.2			3.16 ± 6.8	10.5 ± 7.5

IMA: internal mammary artery; Dys: dyskinesia; Aki: akinesia; Dop: dopamine; Bl: bleeding; Pf: pulmonary failure; Rf: renal failure. Clamp: for grafting or for IMA only.

**Table 3:** Pre and post-operative LV volumes and EF

Pt	BSA m^2^	Nyha pre	Nyha post	ESV pre	ESV post	EDV pre	EDV post	EF% pre	EF% post	Follow up (m)
1	1.7	2	1	102	59	170	119	40	50	60
2	1.7	2	2	72	53	132	112	45	52	51
3	1.8	1	1	74	41	136	84	46	51	48
4	1.8	3	1	106	78	162	130	34	40	48
5	1.8	3	2	83	58	151	102	45	43	47
6	1.8	2	1	106	69	163	126	35	45	43
7	1.7	4	1	82	57	128	134	36	57	30
8	1.7	3	1	95	54	167	129	43	58	27
9	1.7	3	1	101	53	168	119	39	55	15
10	1.9	3	2	100	59	159	123	37	52	13
11	2	2	1	105	55	175	100	40	45	12
12	1.5	2	1	97	57	176	105	44	46	9
mean ± sd	1.7 ± 0.1	2.5 ± 0.8	1.2 ± 0.4	93.5 ± 12.4	57.8 ± 8.9	157.2 ± 16.7	115.3 ± 14.9	40.3 ± 4.2	49.5 ± 5.7	34 ± 18
P val			.001		.001		.001		.001	

Pre: preoperative, post: postoperative.

## Discussion

### Function of the patches

The sandwich double-patch technique has already been used for the treatment of postinfarction ventricular septum rupture [4-6]. The function of the patches is to close the defect with as little tension as possible on the tissue because a direct suture would impose too great a strain on the wall. In the septum rupture the sandwich technique was used to slacken and loosen the tension on the recently infarcted miocardium. In others reports of the technique, always related to interventricular septum rupture, the patches start from the sandwiched septum but diverge towards the inferior free walls of both ventricles [4]. In these cases the role of the patches is to become the inferior walls of left and right ventricles.

The sandwich double-patch technique (briefly "bi-patch closure") has never been described for reconstruction after LVA excision. In our technique the patches have a completely different function from these described above for septal rupture. After LVA excision in our patients the patches have to pull the tissue hard, restrain and stretch the edges of the walls. They have to reduce as much as possible the wide ventricular mouth by a high centripetal tension and traction on the sutures. The external patch acts as a pulley for each stitch when it is pulled and tied, and all together the pulleys draw the edges of the walls towards the centre of the ventricular mouth (Figure 1). Afterwards, the patches work together to support the tight radial stitches and fasten the walls like in the jaws of a vice. Most significantly, bi-patch closure allows the cavity to narrow and closes the gap without felt strips and eversion of the thick edges.

Thick wall edges after LVA resection can be found when a muscular LVA is treated or when a thick border zone is cut down away from the scar. Like other researchers [7] we use digital palpation of the beating walls, which is of pivotal importance to guide excision. Akinesia could be due to segmental afterload mismatch, which disappears during unloaded opened ventricle if the myocardium is still viable. Persisting akinesia of infarcted unloaded segments suggests that they would never contract in the future and they are lost forever. Thus digital palpation can be considered a helpful viability test and it guarantees the correct excision of the non-contracting muscle.

From a mechanical point of view there is an interaction between the patches themselves and the respective neighbouring subendocardial and subepicardial myocardial layers. The edge of a thick cut wall shows two rims: an internal endocardial and an external epicardial rim. Closure of the hole with a single patch only would leave the opposite rim free to be pulled by the respective neighbouring myocardial layer towards the base of the heart and its contraction would become less effective. Closure by two patches allows each of them to become the extension of the respective neighbouring myocardial layer. Both the contractile layers can have a point of rest supporting the tension and both can better contribute to pressure development (Figure 1). Bi-patch closure resizes and reshapes the LV properly converging the walls without purse string and crumpling sutures. Finally, it avoids bending and folding the excluded overlying tissues, as in the case of the wall exclusion procedures (Figure 3). We have never applied this technique to a postero-inferior LVA, which is very rare in our experience.

### Shape and size of the patches

Bi-patch closure is performed by tailoring the patches in a variety of shapes. They have to imitate the excised area, only with a reduction in size. The resected area is usually oval so that circular patches can correct the ventricular elongation and apical deformation which occur during the remodeling process [8]. Others addressed this issue by restraining the mouth of the aneurysm with a purse string suture which also makes the mouth circular [9], or by a single endoventricular patch [2], an inverted T closure [10] or a restraining linear closure [11]. As the conical LV architecture is designed for the twisting of the apex and spreading effect on the blood, some authors have outlined the importance of maintaining the short-axis/long-axis ratio (sphericity index)[12] or the LV conical shape [13-15]. To achieve this, we sometimes used oval or cookie-shaped patches instead of circular patches. The best postoperative conical shape was achieved in 5 patients with septal extension of the scar, where a further treatment was carried out before inserting the patches. The treatment of the septal scar will be described in the future.

The size of the patches depends on the width of the ventricular mouth. Despite the strong traction of the stitches on the patches, the convergence of the edges towards the centre cannot be complete and a residual hole persists and must be closed. Previous experience with the Jatene technique (i.e. crumpling the ventricular mouth by concentric purse string sutures and closure with single internal patch buttressed with external teflon felt strips) [9] taught us that purse string sutures could be pulled so that a mouth of about 6 × 4 cm could be reduced to a hole of 2 cm in diameter (Figure 2, 3). The inner patch is smaller than the external patch because the radius of the endocardial curvature is smaller and stitches have to diverge like sunrays (Figure 1).

EF improved in all patients – it could be due to CABG in 10 patients – but we know it is not a reliable indicator of LV function in volume reduction surgery [16]. More importantly, all patients obtained a satisfactory volume reduction and postoperative LV shape (Figure 4) and they all are doing well at follow-up. LV function probably is not better than the one obtained using other techniques of reconstruction. Bi-patch closure is not designed to replace other well-established procedures, and we did not plan a comparative study. We are still using Cooley and Dor procedures and it is not possible to judge which is the best among their procedures and ours. We only used bi-patch closure to resolve some technical problems in selected cases. Indeed Sponitz said that no formula or procedure will predict the optimum operation for a given dysfunctional area and that clinical judgment and experience are the best guides to management of the individual case [17].

## Conclusion

The sandwich double-patch technique, or bi-patch closure, was already used to slacken and loosen the tension of the sutures in postinfarction IVS rupture. We first adopted it to pull and restrain the edges of the walls after resection of an LVA, to narrow the cavity and close the gap, with good short and long-term results. The technique offers some advantages and does not result in increased morbidity and mortality.

## Competing interests

We wish to thank Sorin Group, Mirandola, Italy for their financial support of the publication.

## Authors' contributions

ET performed all the operations with this technique and made the conception and design of the study. MZ made a critical analysis and interpretation of the data and approved the technique. VF, NP, JM, AN were members of the surgical team in all operations. They took care of the patients in the ward and they participated in the acquisition of clinical, surgical, echocardiographic and follow-up data. NP performed the statistical analysis. All authors read and approved the final manuscript.
